# New insights into the association between *AXIN2* 148 C/T, 1365 C/T, and rs4791171 A/G variants and cancer risk

**DOI:** 10.1186/s12935-019-0840-z

**Published:** 2019-05-06

**Authors:** Bin Xu, Wei Yuan, Li Shi, Li Zuo, Xing-Yu Wu, Wei Zhang, Qiaxian Wen

**Affiliations:** 1 Department of Oncology, Affiliated Hospital of Jiangnan University, Wuxi, 214000 Jiangsu China; 2 Department of Cardiology, Taizhou People’s Hospital, Taizhou, 225300 Jiangsu China; 3 Department of Urology, The Affiliated Changzhou No. 2 People’s Hospital of Nanjing Medical University, Changzhou, 213003 Jiangsu China; 4 Department of Oncology, Taizhou People’s Hospital, 210 Yingchun Road, Taizhou, 225300 Jiangsu China

**Keywords:** *AXIN2*, Polymorphism, Cancer, Analysis, In silico

## Abstract

**Background:**

Many epidemiological studies have investigated association of *AXIN2* variants on overall cancer risks; however, the available results remain inconsistent.

**Methods:**

An updated analysis was conducted to ascertain a more accurate estimation of the correlation between *AXIN2* 148 C/T, 1365 C/T, and rs4791171 A/G polymorphisms and cancer risk. We also used in silico tools to assess the effect of *AXIN2* expression on cancer susceptibility and overall survival time.

**Results:**

A total of 4281 cases and 3955 control participants were studied. The overall results indicated that *AXIN2* 148 C/T variant was associated with cancer risk (allelic contrast: OR = 0.88, 95% CI 0.77–0.99, *P*_heterogeneity_ = 0.004; dominant model: OR = 0.82, 95% CI 0.69–0.96, *P*_heterogeneity_ = 0.022), especially for lung and prostate adenocarcinoma. Similar results were observed in 1365 C/T polymorphism (OR = 0.71, 95% CI 0.61–0.98, *P*_heterogeneity_ = 0.873; dominant model: OR = 0.66, 95% CI 0.47–0.94, *P*_heterogeneity_ = 0.775). Moreover, in subgroup analysis by ethnicity, similar findings were obtained for Asian and Caucasian populations. Results from in silico tools suggested that *AXIN2* expressions in lung adenocarcinoma were lower than that in normal group.

**Conclusions:**

Our findings indicated that *AXIN2* 148 C/T and 1365 C/T variants may be associated with decreased cancer susceptibility.

## Background

The continuing changes in global population and epidemiology indicate that the burden of cancer will continue to increase in the coming decades. Cancer is considered as a multifactorial disease and its occurrence is associated with several factors such as lifestyle, environment and single nucleotide polymorphism (SNP) [1–3]. With the remarkable development of a series of genotyping technologies including genome-wide association studies (GWAS), our understanding of genetic factors related to carcinogenesis has substantially expanded [4–6]. Wnt/β-catenin signaling pathway is known to play a central role in the process of embryogenesis, and abnormalities of this pathway are associated with numerous human malignant tumors [7, 8]. Axin2 protein acts as a negative regulator of Wnt pathway and plays a crucial role in cell differentiation, migration, cytometaplasia, and apoptosis [9–11]. Axin2 protein is also involved in down-regulation of β-catenin translocation ito the nucleus. In this process, Axin2 binds to transcription factors and subsequently inhibits the expression of numerous target genes including *vascular matrix metalloproteinases* (*MMP*), *cox 2*, and *endothelial growth factor* (*VEGF*) [12, 13].

Mutations of *AXIN2* gene has been identified by previous genotyping technologies. This gene is located on human chromosome 17q23-q24 and composed of 10 exons, which encodes a protein consisting of 843 amino acids [14]. Loss of heterozygosity of this gene was previously identified in a number of carcinomas such as hepatoblastoma, hepatocellular carcinoma, melanoma, gastrointestinal, ovarian, synchronous endometrial carcinomas [15–18]. Association between AXIN2 variants and carcinoma susceptibility has also been reported by previous publications. These SNPs including: 148 C>T (rs2240308), 1365 C/T (rs9915936), and rs4791171 A/G (NC_000017.10) [19–24]. Study population of these genetic variants has involved numerous ethnicities such as Brazilians, Iranians, Chinese, Saudi Arabians, Indians and Poles [20–27]. These studies also evaluated various malignancies; nevertheless, there were ambiguous conclusions on the relationship between the *AXIN2* polymorphisms and cancer risk among different case–control studies.

For *AXIN2* 148 C>T polymorphism, a case–control study observed no statistically significant correlation between controls and prostate adenocarcinoma in Turkish population [27]. However, another two studies identified notable decreased risks in Iranian colorectal cancer subjects and Chinese prostate adenocarcinoma participants [21, 22]. Therefore, a meta analysis with all eligible data based on the inclusion criteria was conducted to further assess the associations between *AXIN2* 148 C/T, 1365 C/T, and rs4791171 A/G polymorphisms and cancer risk [19–33].

## Materials and methods

### Literature retrieval strategy

PubMed, Web of Science, Google Scholar, and China Wanfang Databases were systematically searched to identify all eligible published articles on *AXIN2* variants and cancer susceptibility. The following terms were utilized for searching abstracts and titles: “Axin OR *AXIN2*”, “polymorphism OR SNP OR variant”, and “cancer OR adenocarcinoma OR carcinoma OR tumor”. The latest search was conducted on Jan 31, 2019 with no language restrictions. Furthermore, we also carefully screened and manually searched the review or original publications for more eligible studies.

### Study selection

Two authors independently chose the eligible studies based on the inclusion criteria: (a) case–control studies that evaluated the association between *AXIN2* 148 C/T, 1365 C/T, and rs4791171 A/G variants and cancer risk; (b) studies that involved available information for measuring odds ratio (OR) with 95% confidence intervals (CIs); (c) genotype distribution in controls must be conformed to Hardy-Weinberg equilibrium (HWE).

### Data extraction

All related information was independently screened by two investigators (L Shi and B Xu) from each enrolled study, including the name of first author, year of publication, country of origin, ethnicity, source of control, genotyping method, cancer type, total number of participants, *P* value for HWE, age range, genotyping data of *AXIN2* 148 C/T, 1365 C/T, and rs4791171 A/G variants in cases and controls. Disagreement should be resolved by discussion with a third author (W Zhang). If the controversial content still existed, it should be addressed by all investigators to reach a consensus.

### Statistical analysis

The strength of the relationship between *AXIN2* 148 C/T, 1365 C/T, and rs4791171 A/G polymorphisms and cancer susceptibility was measured by calculating OR with 95% CI. A total of four genetic models were adopted in the current analysis, including allelic comparison model (M-allele vs. W-allele), homozygote contrast model (MM vs. WW), heterozygote model (MW vs. WW), and dominant model (MM + MW vs. WW). The χ^2^-test-based *Q* test was performed to investigate *P* value for heterogeneity among eligible researches. If *P* < 0.05, indicating that a significant heterogeneity was found, we employed the random-effects model (DerSimonian–Laird method) [34]. On the other hand, the fixed-effects model (Mantel–Haenszel method) was carried out [35]. We adopted qualitative funnel plot to assess possible publication bias by calculating the standard error of log(OR) for each research plotted against its log(OR). We further conducted quantitative Egger’s test to evaluate funnel plot asymmetry [36]. The web-based program was applied to check for deviations from the Hardy–Weinberg equilibrium (HWE) of distribution frequencies (http://ihg2.helmholtz-muenchen.de/cgibin/hw/hwa1.pl) [37]. The *P* value more than 0.05 suggested an HWE balance. Moreover, we applied leave-one-out sensitivity analyses to calculate the stability of pooled results [38]. All of the above analyses were conducted by STATA software v11.0 (Stata Corporation, TX).

### In silico analysis of *AXIN2* expression

An online gene expression database was adopted to investigate the *AXIN2* expression in lung and prostate adenocarcinoma tissues and the paracancerous tissues. (http://gemini.cancer-pku.cn/) [39]. RNA expression profiles of 446 pathologically diagnosed lung adenocarcinoma (including 387 Caucasians, 51 African-Americans, and 8 Asians) and 153 prostate adenocarcinoma tissues (containing 147 Caucasians and 6 African-Americans) were evaluated by this database. The Cancer Genome Atlas (TCGA) samples were also utilized to investigate the high and low expression of *AXIN2* on cancer susceptibility and overall survival time. Moreover, the String online server was applied to assess the gene–gene correlation of *AXIN2* (http://string-db.org/).

## Results

### Characteristics of studies

**Fig. 1:**
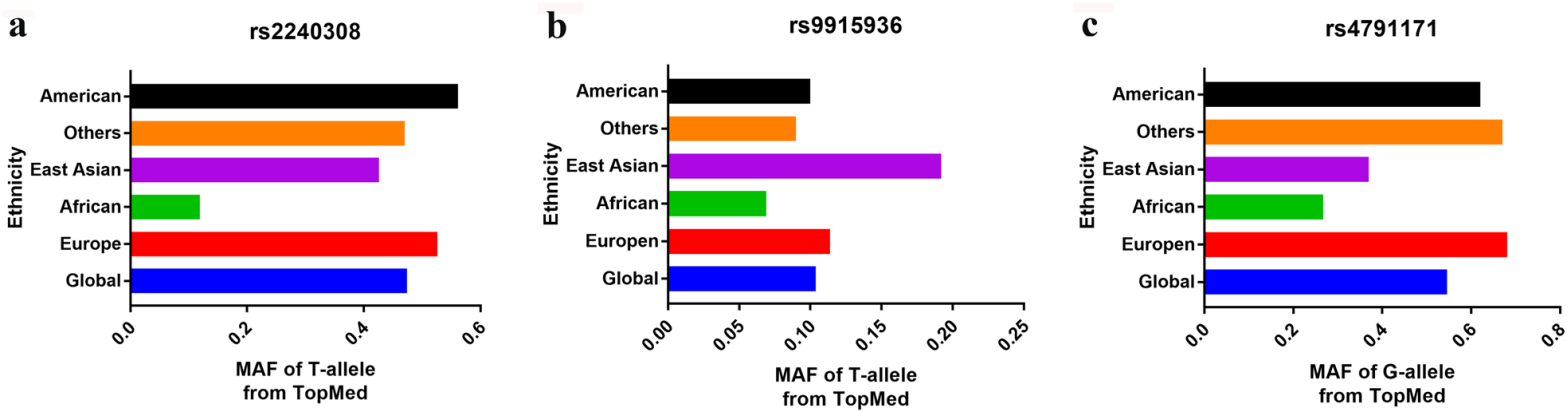
Minor allele and major allele frequencies for *AXIN2* 148 C/T (**a**), 1365 C/T (**b**), and rs4791171 A/G (**c**) variants in controls stratified by ethnicity. Vertical line, allele frequency; Horizontal line, allele type

As was shown in Table 1, 15 articles were finally retrieved in the present analysis, which contains 22 case–control studies for *AXIN2* 148 C/T, 1365 C/T, and rs4791171 A/G variants. There were 2909 cancer subjects and 2907 control volunteers for 148 C/T polymorphism, 587 cancer subjects and 605 controls for 1365 C/T variant, 785 cases and 443 controls for rs4791171 A/G variant. Furthermore, we checked the minor allele frequencies (MAF) of three *AXIN2* variants by Trans-Omics for Precision Medicine (TOPMed) online (https://www.ncbi.nlm.nih.gov/snp/) (Fig. 1). MAF of *AXIN2* 148 C/T were: in Africans, 0.119; Asians, 0.426; Europeans, 0.526; Americans, 0.561; others (including Pacific Islanders), 0.470; Global, 0.474. MAF of *AXIN2* 1365 C/T were: in Africans, 0.069; East Asians, 0.192; Europeans, 0.114; Americans, 0.100; others, 0.090; Global, 0.104. Finally, MAF of *AXIN2* rs4791171 A/G were: in Africans, 0.267; East Asians, 0.370; Europeans, 0.681; Americans, 0.620; others, 0.670; Global, 0.547. In stratified analysis by ethnicity, seven studies were performed in Caucasian populations, twelve studies were in Asian descendants, and two were done in Arabians and one was in Latin descendants. Eight studies were conducted using population based controls and the rest 14 studies were utilizing hospital based controls. The classical genotyping method, PCR-restriction fragment length polymorphism (RFLP) was adopted in nine of these studies.

**Table 1:** Basic information for included studies of the correlation between *AXIN2* 148 C/T, 1365 C/T, and rs4791171 A/G variations and cancer risk

Author/year	Origin	Ethnicity	Source	Cancer	Method	Age range	Age range	Case	Control	Case			Control			HWE
148 C/T						Case	Control			TT	TC	CC	TT	TC	CC	
Kanzaki 2006 [26]	Japan	Asian	PB	LC	PCR–RFLP	66.4 (mean)	NA	160	109	8	71	81	15	52	42	0.863
Kanzaki 2006 [26]	Japan	Asian	PB	HNC	PCR–RFLP	66.4 (mean)	NA	63	109	9	29	25	15	52	42	0.863
Kanzaki 2006 [26]	Japan	Asian	PB	CRC	PCR–RFLP	66.4 (mean)	NA	113	109	15	44	54	15	52	42	0.863
Gunes 2009 [19]	Turkey	Caucasian	PB	LC	PCR	59.22 ± 9.63	57.01 ± 7.89	100	100	8	47	45	16	52	32	0.501
Gunes 2010 [25]	Turkey	Caucasian	HB	AT	PCR	58.66 ± 8.04	57.01 ± 7.89	100	100	16	45	39	16	52	32	0.501
Pinarbasi 2011 [27]	Turkey	Caucasian	HB	PC	PCR	70.38 ± 7.78	68.55 ± 4.47	84	100	19	35	30	18	48	34	0.883
Naghibal 2012 [21]	Iran	Asian	HB	CRC	PCR–RFLP	NA	NA	110	179	19	57	34	26	98	55	0.096
Liu 2014 [28]	China	Asian	PB	LC	RT-PCR	57.78 ± 9.89	52.21 ± 10.56	498	533	47	216	235	67	255	211	0.457
Ma 2014 [22]	China	Asian	PB	PC	PCR	71.2 (mean)	70.4 (mean)	103	100	11	31	61	9	52	39	0.153
Mostowska 2014 [24]	Poland	Caucasian	HB	OC	PCR–RFLP	58.4 ± 9.7	57.4 ± 7.5	228	282	46	115	67	65	146	71	0.546
Yadav 2016 [23]	India	Asian	HB	GBC	Taqman	52.05 ± 10.06	43.2 ± 11.5	564	250	119	253	192	44	108	98	0.138
Liu 2016 [32]	China	Asian	HB	PTC	MassARRAY	45.13 ± 10.97	41.9 ± 10.22	53	50	2	24	27	4	29	17	0.084
Kim 2016 [31]	Korea	Asian	HB	HCC	Goldengate	53.8 ± 10.3	41.1 ± 10.3	242	482	18	100	124	41	195	246	0.789
Rosales 2016 [29]	Mexico	Latin	PB	CRC	PCR–RFLP	59.03 (mean)	36.88 (mean)	188	99	54	109	25	18	59	22	0.054
Bahl 2017 [30]	India	Asian	HB	LC	PCR–RFLP	57.38 ± 10.74	53.23 ± 10.44	303	305	54	150	99	80	144	81	0.330

1365 C/T										TT	TC	CC	TT	TC	CC	
Bahl 2017 [30]	India	Asian	HB	LC	PCR–RFLP	57.38 ± 10.74	53.23 ± 10.44	303	305	6	29	268	5	51	249	0.215
Pinarbasi 2011 [27]	Turkey	Caucasian	HB	PC	PCR	70.38 ± 7.78	68.55 ± 4.47	84	100	0	7	77	0	8	92	0.677
Gunes 2010 [25]	Turkey	Caucasian	HB	AT	PCR	58.66 ± 8.039	57.01 ± 7.89	100	100	0	9	91	0	12	88	0.523
Gunes 2009 [19]	Turkey	Caucasian	PB	LC	PCR	59.22 ± 9.63	57.01 ± 7.89	100	100	0	9	91	0	12	88	0.523

rs4791171A/G										GG	GA	AA	GG	GA	AA	
Alanazi 2013 [20]	Saudi	Arabian	HB	BC	RT-PCR	48.0 (mean)	NA	99	83	21	44	34	17	44	22	0.559
Yadav 2016 [23]	India	Asian	HB	GBC	PCR–RFLP	52.05 ± 10.06	43.2 ± 11.5	564	250	228	248	88	97	118	35	0.926
Parine 2019 [33]	Saudi	Arabian	HB	CRC	TaqMan	57.0 (mean)	NA	122	110	27	55	40	24	48	38	0.236

*HB* hospital-based, *PB* population-based, *AT* astrocytoma, *BC* breast cancer, *CRC* colorectal cancer, *GBC* gallbladder cancer, *PCR–RFLP* polymerase chain reaction and restrictive fragment length polymorphism, *RT* real time, *NA* not available, *NOS* Newcastle–Ottawa Scale, *HCC* hepatocellular carcinoma, *HNC* head and neck cancer, *HWE* Hardy–Weinberg equilibrium of controls, *LC* lung adenocarcinoma, *PC* prostate adenocarcinoma, *PTC* papillary thyroid carcinoma, *OC* ovarian cancer

### Quantitative synthesis

**Fig. 2:**
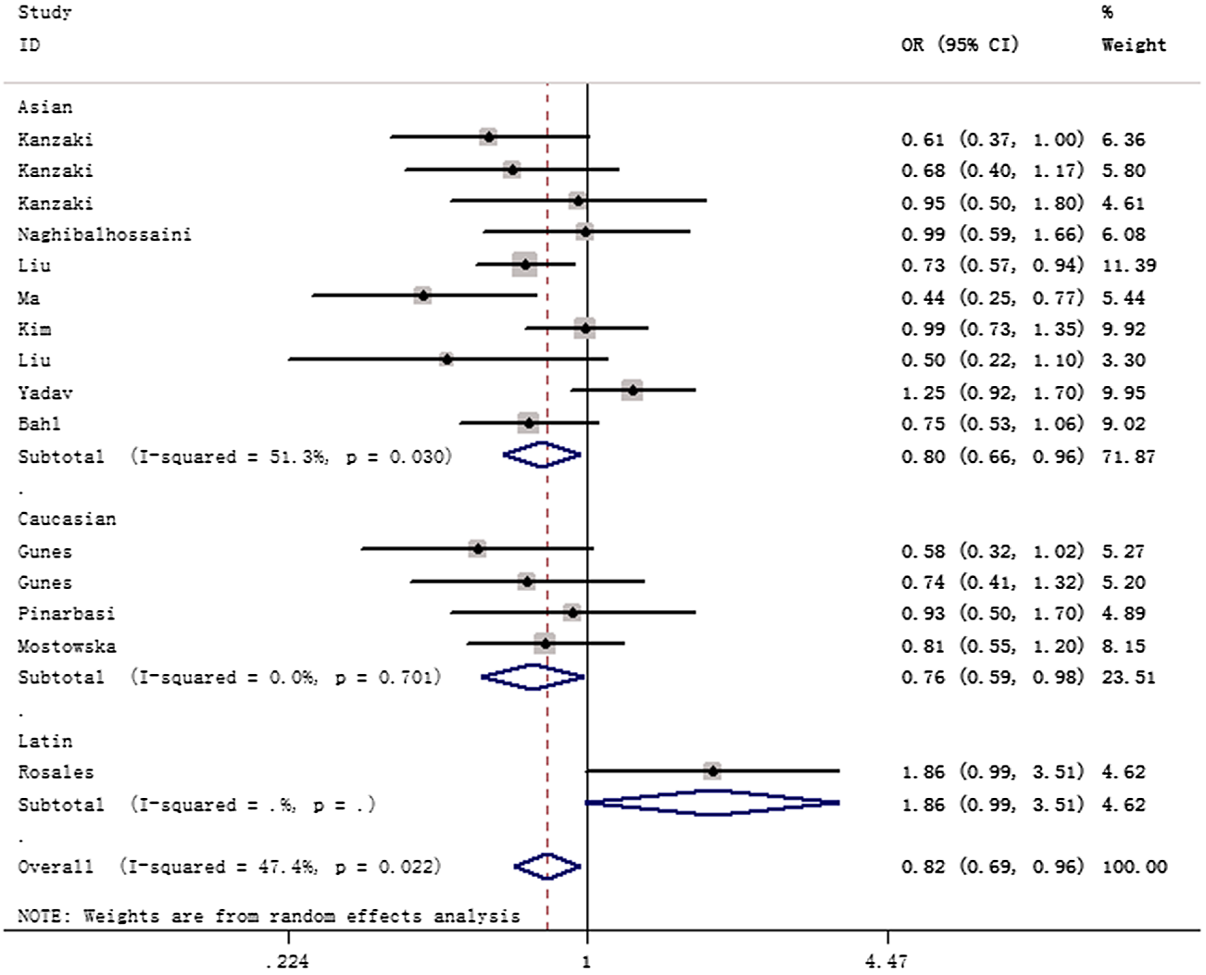
Forest plot of cancer susceptibility correlated with *AXIN2* 148 C/T variant (heterozygote comparison of TC vs. CC, fixed-effects) in the stratified analyses by ethnicity

**Fig. 3:**
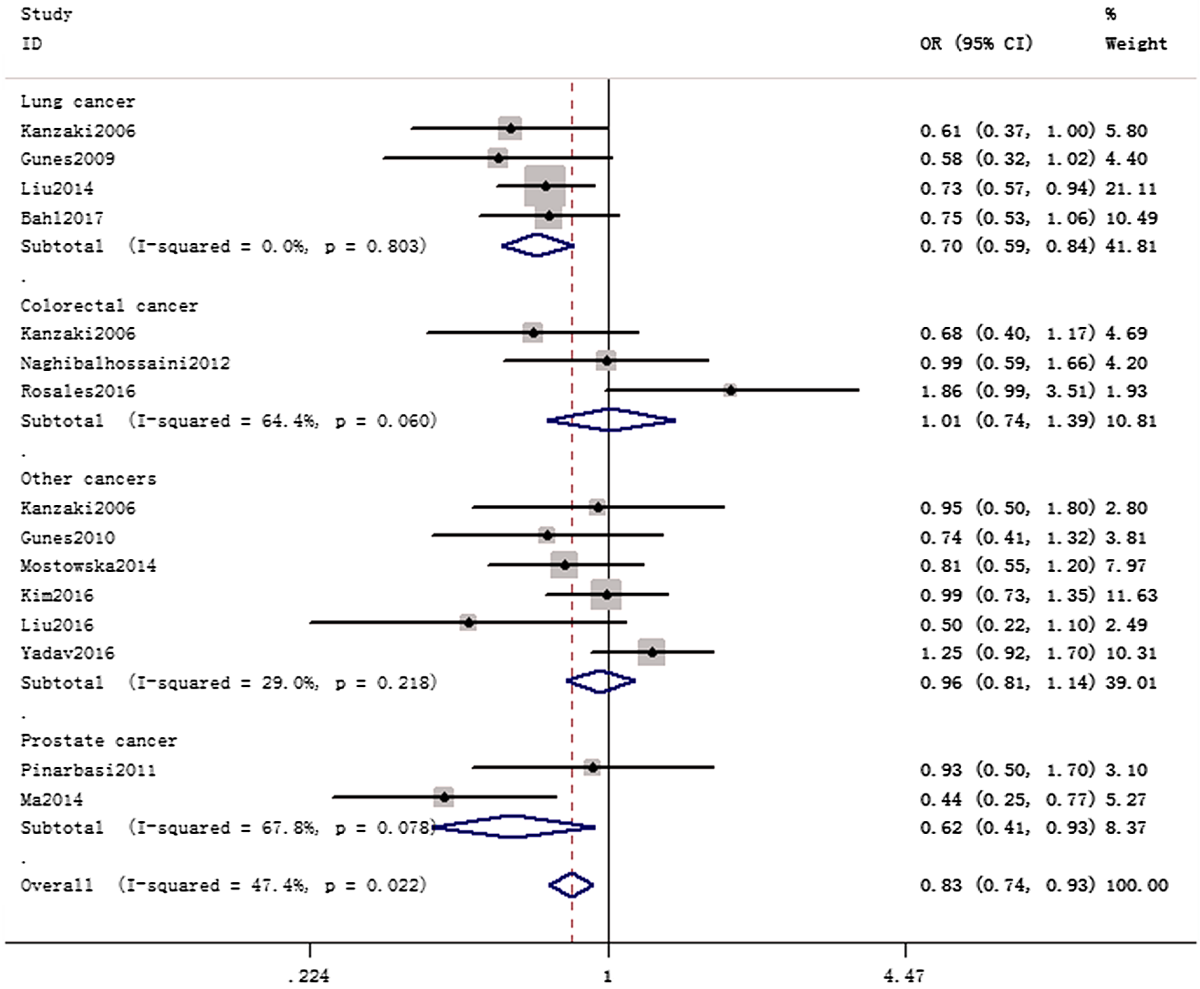
Forest plot of TC versus CC genetic model of *AXIN2* 148 C/T polymorphism in the stratified analyses by cancer type (fixed-effects)

In the overall analysis, we identified a significant correlation between *AXIN2* 148 C/T variant and cancer risk (allele contrast: OR = 0.88, 95% CI 0.77–0.99, *P*_heterogeneity_ = 0.004, *P* = 0.041; heterozygote comparison: OR = 0.84, 95% CI 0.75–0.95, *P*_heterogeneity_ = 0.112, *P* = 0.004; dominant genetic model: OR = 0.82, 95% CI 0.69–0.96, *P*_heterogeneity_ = 0.022, *P* = 0.015) (Table 2). In subgroup analysis by race, we observed positive results in Asians (allele contrast: OR = 0.85, 95% CI 0.73–0.98, *P*_heterogeneity_ = 0.016, *P* = 0.027; dominant genetic model: OR = 0.80, 95% CI 0.66–0.96, *P*_heterogeneity_ = 0.030, *P* = 0.020) and Caucasians (dominant genetic model: OR = 0.76, 95% CI 0.59–0.98, *P*_heterogeneity_ = 0.701, *P* = 0.036), (Fig. 2). Moreover, subgroup analysis by cancer type suggested that 148 C/T variant was associated with a decreased cancer risk in lung adenocarcinoma (allele contrast: OR = 0.74, 95% CI 0.65–0.84, *P* value for heterogeneity = 0.602, *P* < 0.001; dominant genetic model: OR = 0.70, 95% CI 0.59–0.84, *P*_heterogeneity_ = 0.803, *P* < 0.001, Fig. 3). Similar finding was indicated in prostate adenocarcinoma (heterozygote comparison: OR = 0.54, 95% CI 0.35–0.84, *P*_heterogeneity_ = 0.088, *P* = 0.006; dominant genetic model: OR = 0.62, 95% CI 0.41–0.93, *P*_heterogeneity_ = 0.078, *P* = 0.022). In subgroup analysis by source of control, similar results were also observed in population-based studies. Furthermore, we identified notable correlation between *AXIN2* 1365 C/T variant and cancer risk (allele contrast: OR = 0.71, 95% CI 0.61–0.98, *P*_heterogeneity_ = 0.873, *P* = 0.038; heterozygote comparison: OR = 0.63, 95% CI 0.44–0.91, *P*_heterogeneity_ = 0.668, *P* = 0.014; dominant model: OR = 0.66, 95% CI 0.47–0.94, *P*_heterogeneity_ = 0.775, *P* = 0.021). For rs4791171 A/G polymorphism, no significant association was indicated (allele comparison, OR = 0.99, 95% CI 0.85–1.17, *P*_heterogeneity_ = 0.786, *P* = 0.864; homozygote contrast, OR = 0.94, 95% CI 0.66–1.33, *P*_heterogeneity_ = 0.873, *P* = 0.728; heterozygote contrast, OR = 0.86, 95% CI 0.62–1.17, *P*_heterogeneity_ = 0.522, *P* = 0.322; dominant model, OR = 0.89, 95% CI 0.66–1.19, *P*_heterogeneity_ = 0.575, *P* = 0.429).

**Table 2:** Stratified analyses of *AXIN2* 148 C/T, 1365 C/T, and rs4791171 A/G variants on overall cancer risk

Variables	N	Case/control	OR (95% CI)	*P*_h_	*P*	OR (95% CI)	*P*_h_	*P*	OR (95% CI)	*P*_h_	*P*	OR (95% CI)	*P*_h_	*P*
			M-allele vs. W-allele			MM vs. WW			MW vs. WW			MM+MW vs. WW		
148 C/T														
Total	15	2909/2907	0.88 (0.77–0.99)	0.004	0.041	0.82 (0.63–1.06)	0.007	0.132	0.84 (0.75–0.95)	0.112	0.004	0.82 (0.69–0.96)	0.022	0.015
Ethnicity														
Asian	10	2209/2226	0.85 (0.73–0.98)	0.016	0.027	0.76 (0.57–1.03)	0.040	0.078	0.84 (0.74–0.96)	0.074	0.009	0.80 (0.66–0.96)	0.030	0.020
Caucasian	4	512/582	0.85 (0.72–1.01)	0.380	0.061	0.75 (0.53–1.07)	0.304	0.108	0.77 (0.58–1.00)	0.896	0.053	0.76 (0.59–0.98)	0.701	0.036
Latin	1	188/99	1.48 (1.05–2.09)	–	0.026	2.64 (1.21–5.78)	–	0.015	1.63 (0.84–3.13)	–	0.146	1.86 (0.99–3.51)	–	0.054
Cancer type														
LC	4	1061/1047	0.74 (0.65–0.84)	0.602	< 0.001	0.53 (0.40–0.70)	0.360	< 0.001	0.76 (0.63–0.92)	0.865	0.005	0.70 (0.59–0.84)	0.803	< 0.001
CRC	3	411/387	1.10 (0.90–1.35)	0.071	0.348	1.36 (0.87–2.11)	0.096	0.178	0.96 (0.68–1.34)	0.123	0.796	1.01 (0.74–1.39)	0.060	0.932
PC	2	187/200	0.83 (0.62–1.12)	0.099	0.223	1.00 (0.54–1.87)	0.509	0.987	0.54 (0.35–0.84)	0.088	0.006	0.62 (0.41–0.93)	0.078	0.022
Others	7	1250/1273	0.98 (0.87–1.11)	0.217	0.751	0.98 (0.76–1.26)	0.363	0.862	0.96 (0.80–1.15)	0.375	0.640	0.96 (0.81–1.14)	0.218	0.664
Source														
HB	8	1684/1748	0.94 (0.85–1.04)	0.093	0.219	0.89 (0.72–1.09)	0.128	0.267	0.93 (0.80–1.09)	0.564	0.364	0.92 (0.80–1.07)	0.268	0.272
PB	7	1225/1159	0.82 (0.65–1.02)	0.009	0.074	0.73 (0.44–1.22)	0.007	0.235	0.74 (0.62–0.88)	0.083	0.001	0.74 (0.56–0.97)	0.037	0.032
1365 C/T														
Total	4	587/605	0.71 (0.61–0.98)	0.873	0.038	1.11 (0.34–3.70)	–	0.859	0.63 (0.44–0.91)	0.668	0.014	0.66 (0.47–0.94)	0.775	0.021
Ethnicity														
Asian	1	303/305	0.66 (0.43–0.99)	–	0.043	1.11 (0.34–3.70)	–	0.859	0.53 (0.32–0.86)	–	0.010	0.58 (0.37–0.92)	–	0.020
Caucasian	3	284/300	0.81 (0.47–1.38)	0.855	0.440	NA			0.80 (0.46–1.39)	0.846	0.428	0.80 (0.46–1.39)	0.846	0.428
Cancer type														
LC	2	403/405	0.67 (0.46–0.97)	0.806	0.034	1.11 (0.34–3.70)	–	0.859	0.57 (0.37–0.87)	0.548	0.009	0.61 (0.41–0.91)	0.669	0.016
PC	1	84/100	1.04 (0.37–2.94)	–	0.936	NA			1.05 (0.36–3.01)	–	0.934	1.05 (0.36–3.01)	–	0.934
AT	1	100/100	0.74 (0.30–1.79)	–	0.503	NA			0.73 (0.29–1.81)	–	0.490	0.73 (0.29–1.81)	–	0.490
rs4791171 A/G														
Total	3	785/443	0.99 (0.85–1.17)	0.786	0.864	0.94 (0.66–1.33)	0.873	0.728	0.86 (0.62–1.17)	0.522	0.322	0.89 (0.66–1.19)	0.575	0.429
Ethnicity														
Asian	1	564/250	1.00 (0.80–1.24)	–	0.997	0.93 (0.69–1.48)	–	0.773	0.84 (0.63–1.31)	–	0.434	0.88 (0.68–1.34)	–	0.556
Arabian	2	221/193	0.96 (0.73–1.27)	0.511	0.778	0.96 (0.66–1.62)	0.603	0.843	0.87 (0.56–1.36)	0.257	0.538	0.89 (0.69–1.36)	0.294	0.596
Cancer type														
BC	1	99/83	0.87 (0.57–1.31)	–	0.497	0.80 (0.35–1.84)	–	0.599	0.65 (0.33–1.28)	–	0.209	0.69 (0.36–1.31)	–	0.255
GBC	1	564/250	1.00 (0.80–1.24)	–	0.997	0.93 (0.59–1.48)	–	0.773	0.84 (0.53–1.31)	–	0.434	0.88 (0.58–1.34)	–	0.556
CRC	1	122/110	1.04 (0.72–1.51)	–	0.822	1.07 (0.53–2.17)	–	0.854	1.09 (0.60–1.96)	–	0.778	1.08 (0.63–1.87)	–	0.777

*AT* astrocytoma, *BC* breast cancer, *CRC* colorectal cancer, *HB* hospital-based, *PB* population-based, *NA* not available, *LC* lung adenocarcinoma, *PC* prostate adenocarcinoma, *GBC* gallbladder cancer

^a^*P* value of *Q*-test for heterogeneity test (*P*_heter_)

### In silico analysis of *AXIN2* expression

**Fig. 4:**
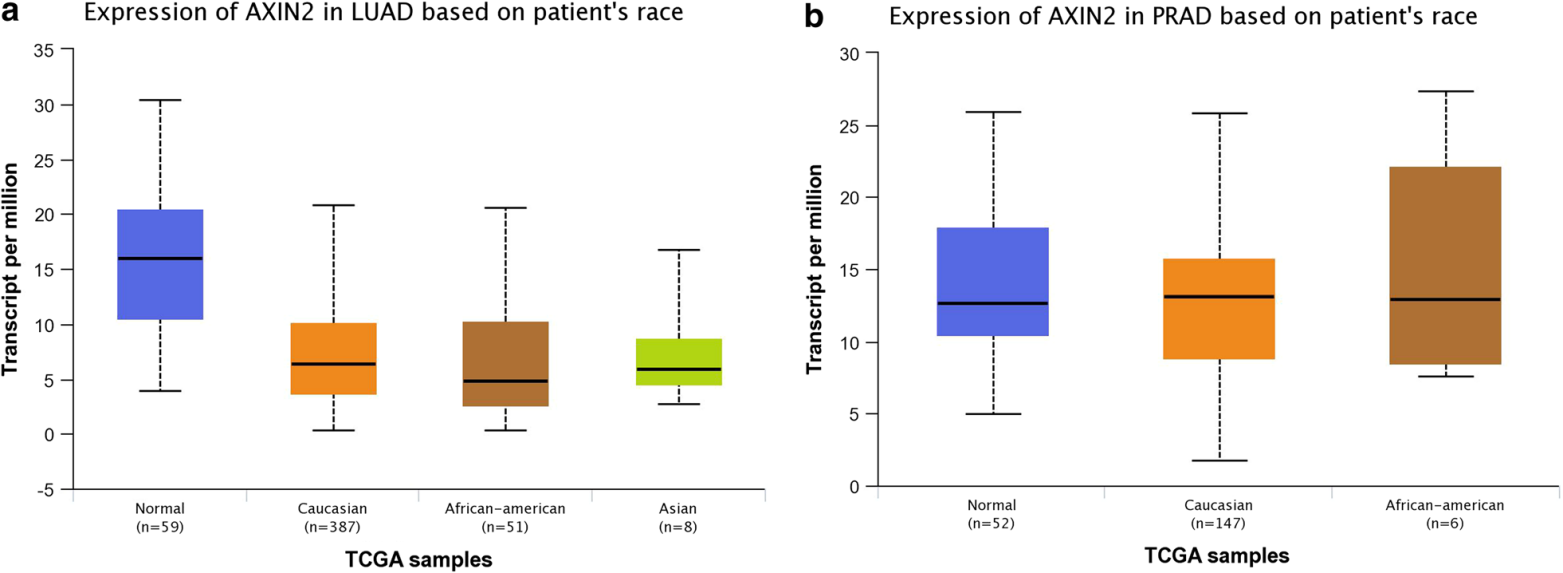
In silico analysis of *AXIN2* expressions in lung adenocarcinoma (**a**) and prostate adenocarcinoma (**b**)

**Fig. 5:**
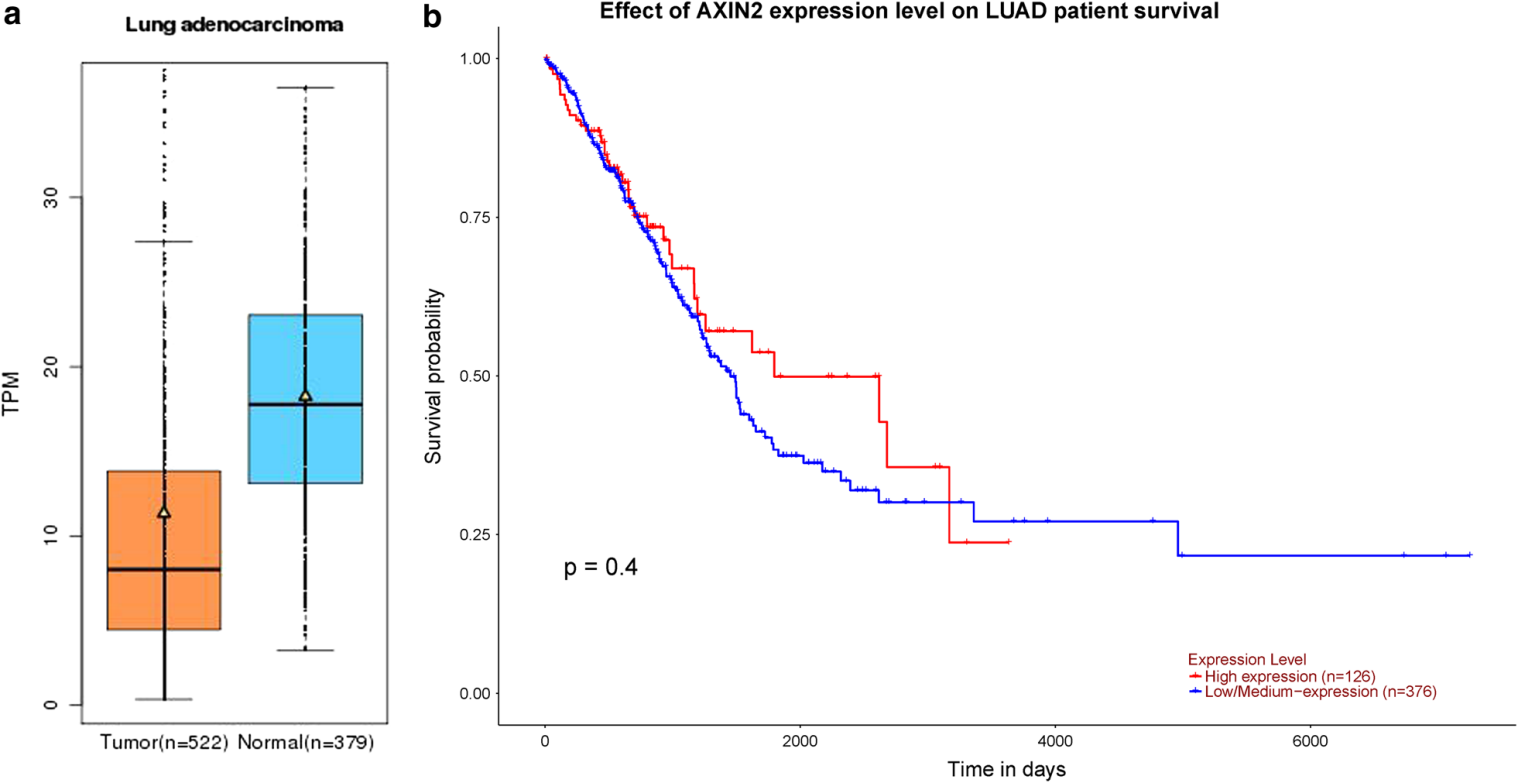
Association of *AXIN2* expression and the overall survival (OS) time among lung adenocarcinoma participants. Expression of *AXIN2* was decreased in lung adenocarcinoma tissue (**a**). However, no vital influence of overall survival time was indicated between high and low *AXIN2* expression groups (**b**, *P* > 0.05)

Results from in silico tools suggested that *AXIN2* expression in normal group was higher than that in lung adenocarcinoma tissue (Fig. 4a). However, no obvious difference was indicated for prostate adenocarcinoma (Fig. 4b). Moreover, we explored whether the *AXIN2* expression had an effect on the overall survival time of lung adenocarcinoma patients. However, Kaplan–Meier estimate showed no vital difference of overall survival time between high and low *AXIN2* expression groups (*P* = 0.40, Fig. 5).

### Publication bias and sensitivity analyses

**Fig. 6:**
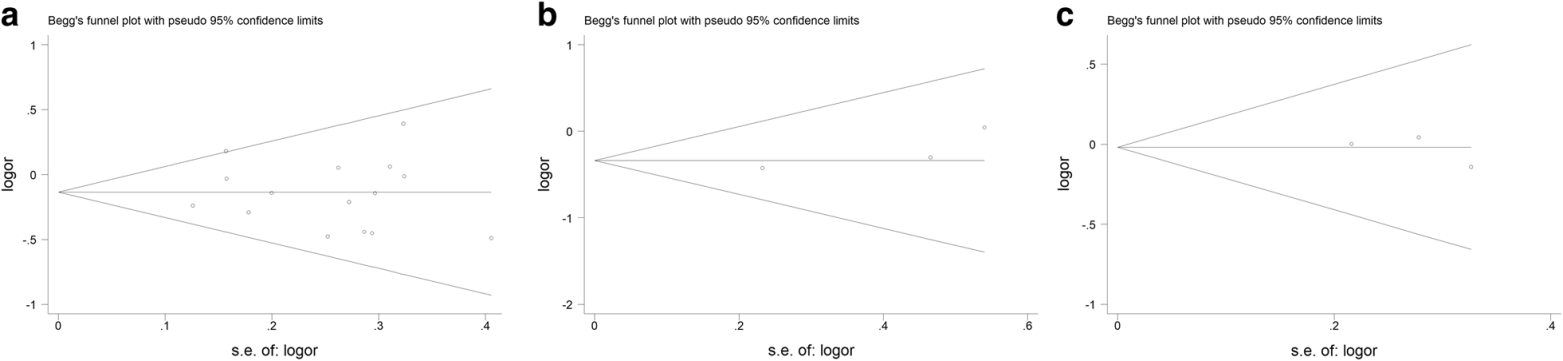
Begg’s funnel plot of publication bias for *AXIN2* 148 C/T (**a**), 1365 C/T (**b**), and rs4791171 A/G (**c**) under dominant model

**Fig. 7:**
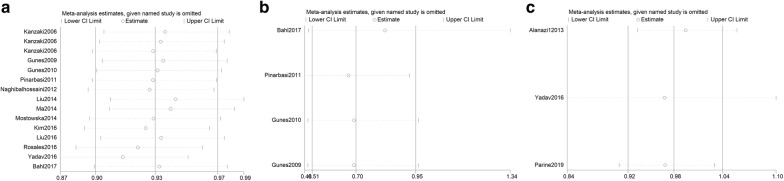
Sensitivity analyses about *AXIN2* 148 C/T, 1365 C/T, and rs4791171 A/G variants and cancer risk (Dominant genetic model of MM + MW vs. WW). Leave-one-out sensitivity analyses were carried out to assess the stability of the overall results. No single study can substantially change the overall OR for *AXIN2* 148 C/T (**a**), 1365 C/T (**b**), and rs4791171 A/G (**c**) polymorphisms

Egger’s test and Begg’s funnel plot were utilized to evaluate publication bias in all of enrolled studies. We demonstrated no publication bias for *AXIN2* 148 C/T polymorphism (allelic contrast, t = − 0.52, *P* = 0.614; TT vs. CC, t = − 0.66, *P* = 0.519; heterozygote comparison, t = − 0.30, *P* = 0.771; TT + TC vs. CC, t = − 0.34, *P* = 0.741), *AXIN2* 1365 C/T variant (allelic comparison, t = 2.20, *P* = 0.159; TC vs. CC, t = 2.18, *P* = 0.161) and rs4791171 A/G polymorphism (G-allele versus A-allele, t = − 0.55, *P* = 0.680; homozygote contrast, t = − 0.62, *P* = 0.645; GA vs. AA, t = − 0.72, *P* = 0.602; dominant model, t = − 0.78, *P* = 0.577). As shown in Fig. 6, results from funnel plots appeared symmetrical in the overall analysis under dominant model, which indicated a lack of publication bias. Sensitivity analyses were also utilized to assess the pooled OR by omission of any one study. The results suggested that the current data from pooled ORs were relatively stable. No single study can substantially change the overall OR (Fig. 7).

## Discussion

To date, large quantities of studies have been conducted to explore whether the variants confer individual’s susceptibility to carcinoma. However, results from the previous publications have yielded controversial results [21, 22]. A previous study based on Indian descendants found a strong protective effect in participants having heterozygous genotype for 1365 C/T variant [30], while another study group did not observe such positive correlation in Turkish population [27]. In 2005, Wu et al. performed a meta-analysis and found that *AXIN2* rs2240308 variant may increase the risk of cancer, especially lung cancer in Asian descendants [40]. Two years later, another meta-analysis indicated no obvious correlation between this variant and cancer risk in the overall analysis. Moreover, researches of this article observed that rs2240308 polymorphism was significantly associated with a decreased cancer risk in Asian population [41]. The overall goal of the present study was to evaluate all eligible data based on the inclusion criteria to enhance the statistical powers and draw more accurate conclusions.

In the current study, a total of 4281 cases and 3955 control participants were investigated. The overall results showed evidence that *AXIN2* 148 C/T variant was associated with decreased cancer risk, especially for lung and prostate adenocarcinoma, which is in line with conclusions identified by Kanzaki et al. Liu et al. and Gune et al. [19, 26, 28]. Similar results were observed in *AXIN2* 1365 C/T polymorphism (under allelic contrast, heterozygote comparison, and dominant genetic model). Moreover, in subgroup analysis by ethnicity, positive findings were obtained for Asian and Caucasian populations. In the stratified analysis by source of control, similar findings were identified in population-based studies for *AXIN2* 148 C/T variant, which is consistent with the findings reported by Yu et al. [41]. Moreover, results from in silico tools showed that *AXIN2* expressions in lung cancer and prostate cancer are lower than that in normal counterpart. High expression of *AXIN2* may have longer OS time than low expression group for lung cancer participants, which were consistent with results derived from the present meta-analysis. Nevertheless, we indicated no significant difference between the high expression and low/medium expression of *AXIN2* in prostate cancer patients.

**Fig. 8:**
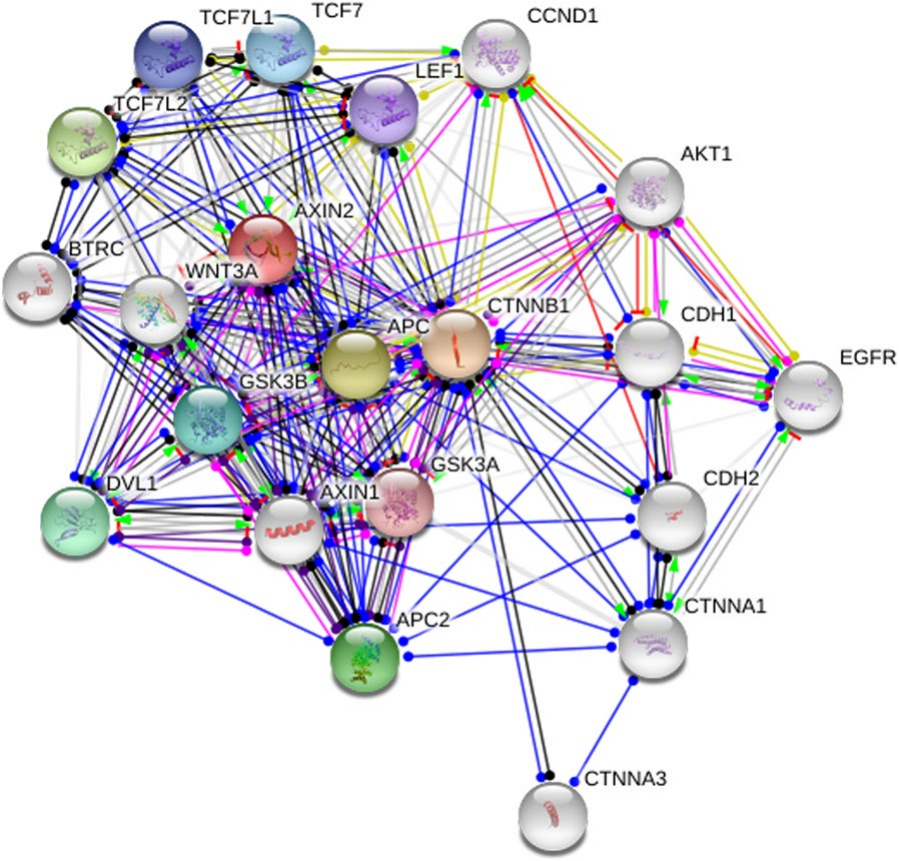
*AXIN2* correlations crosstalk with other genes determined by String server (Homo sapiens). 20 related genes could participate in the gene–gene interaction

Some limitations of the above analysis should be mentioned. Firstly, the numbers of enrolled articles in the current analysis were still not large enough for the comprehensive analysis, especially for *AXIN2* 1365 C/T and rs4791171 A/G variants. Four articles towards *AXIN2* 1365 C/T and three articles for rs4791171 A/G polymorphism were eligible based on the selection criteria. Secondly, insufficient original data from the raw articles limited further evaluation of potential interactions, including relationship between the *AXIN2* 148 C/T, 1365 C/T, and rs4791171 A/G variants and different tumor grade and stage. Thirdly, meta-analysis was based on unadjusted estimates, which may lead to serious confounding bias. Furthermore, gene–gene interaction would also participate in etiological mechanism of carcinoma. As shown in Fig. 8, at least 20 related genes may be involved in such interaction, which are required to be further investigated in future studies. On the other hand, core advantages in current analysis should also be acknowledged. Firstly, a comprehensive study of the correlation of the *AXIN2* 148 C/T, 1365 C/T, and rs4791171 A/G variants with overall cancer susceptibility is statistically more powerful than single case–control study. All the studies according to the inclusion criteria were accumulated in our analysis. Secondly, genotype distribution of controls is conformed to Hardy–Weinberg equilibrium (HWE) in any of the enrolled studies and no significant publication bias was found, which indicated that conclusions of the present analysis are relatively trustworthy.

## Conclusions

Taken together, the current study showed evidence that *AXIN2* 148 C/T and 1365 C/T variants may be associated with decreased cancer susceptibility, especially for lung and prostate adenocarcinoma. Future large scale studies with standardized unbiased cases and well-matched control subjects are needed to ascertain these finding in more detail.

## Abbreviations

AT: astrocytoma
BC: breast cancer
CRC: colorectal cancer
GBC: gallbladder cancer
HB: hospital-based
PB: population-based
PCR-RFLP: polymerase chain reaction and restrictive fragment length polymorphism
RT: real time
NA: not available
NOS: Newcastle–Ottawa Scale
HCC: hepatocellular carcinoma
HNC: head and neck cancer
HWE: Hardy–Weinberg equilibrium of controls
LC: lung adenocarcinoma
PC: prostate adenocarcinoma
PTC: papillary thyroid carcinoma
OC: ovarian cancer
